# Maternal Obesity Reduces Milk Lipid Production in Lactating Mice by Inhibiting Acetyl-CoA Carboxylase and Impairing Fatty Acid Synthesis

**DOI:** 10.1371/journal.pone.0098066

**Published:** 2014-05-21

**Authors:** Jessica L. Saben, Elise S. Bales, Matthew R. Jackman, David Orlicky, Paul S. MacLean, James L. McManaman

**Affiliations:** 1 Division of Basic Reproductive Sciences, University of Colorado Anschutz Medical Campus, Aurora, Colorado, United States of America; 2 Graduate Program in Reproductive Sciences, University of Colorado Anschutz Medical Campus, Aurora, Colorado, United States of America; 3 Center for Human Nutrition, University of Colorado Anschutz Medical Campus, Aurora, Colorado, United States of America; 4 Department of Medicine, Division of Endocrinology, Diabetes and Metabolism, University of Colorado Anschutz Medical Campus, Aurora, Colorado, United States of America; 5 Department of Pathology, University of Colorado Anschutz Medical Campus, Aurora, Colorado, United States of America; 6 Department of Physiology and Biophysics, University of Colorado Anschutz Medical Campus, Aurora, Colorado, United States of America; University of Santiago de Compostela School of Medicine - CIMUS, Spain

## Abstract

Maternal metabolic and nutrient trafficking adaptations to lactation differ among lean and obese mice fed a high fat (HF) diet. Obesity is thought to impair milk lipid production, in part, by decreasing trafficking of dietary and *de novo* synthesized lipids to the mammary gland. Here, we report that *de novo* lipogenesis regulatory mechanisms are disrupted in mammary glands of lactating HF-fed obese (HF-Ob) mice. HF feeding decreased the total levels of acetyl-CoA carboxylase-1 (ACC), and this effect was exacerbated in obese mice. The relative levels of phosphorylated (inactive) ACC, were elevated in the epithelium, and decreased in the adipose stroma, of mammary tissue from HF-Ob mice compared to those of HF-fed lean (HF-Ln) mice. Mammary gland levels of AMP-activated protein kinase (AMPK), which catalyzes formation of inactive ACC, were also selectively elevated in mammary glands of HF-Ob relative to HF-Ln dams or to low fat fed dams. These responses correlated with evidence of increased lipid retention in mammary adipose, and decreased lipid levels in mammary epithelial cells, of HF-Ob dams. Collectively, our data suggests that maternal obesity impairs milk lipid production, in part, by disrupting the balance of *de novo* lipid synthesis in the epithelial and adipose stromal compartments of mammary tissue through processes that appear to be related to increased mammary gland AMPK activity, ACC inhibition, and decreased fatty acid synthesis.

## Introduction

Nutrition during fetal and neonatal periods impacts the risk of developing chronic adult diseases and disorders, such as diabetes and obesity [1]–[4]. Breast milk, which is considered the “gold standard” for neonatal nutrition, has been linked to numerous health benefits for human infants, including reduced risk of developing obesity [5]. It has been reported that maternal obesity can interfere with the ability to initiate and sustain lactation [6], [7], minimizing the potential benefits of breastfeeding for developing infants. Breast milk lipids provide a major source of calories and essential fatty acids necessary for membrane synthesis and neonatal brain development [8]–[10]. Preclinical evidence suggests that high fat (HF) feeding and/or obesity may delay mammary gland development [11], [12], and reduce the transfer of essential fatty acids to offspring through milk [3], [9], [13]. Consequently, diet- or obesity-induced defects in lactation and/or milk lipid production pose potential risks for growth or developmental abnormalities in breastfed offspring.

Milk lipid synthesis is a tightly regulated process [14], [15]. Although it is highly variable among species, within a given species the total concentration of milk lipids is thought to be relatively constant under normal conditions, if nutrition is adequate [14]. Previous studies have shown that the fatty acid composition of milk lipids is influenced by the content and composition of dietary fat [3], [14], and both dietary and *de novo* synthesized fatty acids are found in milk lipids [3], [14]. In mice, there is a progressive increase in the amounts of *de novo* synthesized fatty acids in milk lipids during lactation [16], suggesting that the *de novo* fatty acid synthesis pathway is regulated, in part, by the nutritional demands of growing neonates. There is also increasing evidence in rodent models that high fat diet-induced maternal obesity can alter milk lipid concentration [11], [13], [17]–[19], suggesting that, in addition to neonatal demand, milk lipid production may be influenced by dietary factors and/or maternal metabolism. However, few details are known about the nature of these factors, or how they impact milk lipid formation.

The potential of both diet and obesity to influence milk lipids complicates efforts to understand the effects of maternal obesity on milk properties, and the possible contributions of maternal metabolism to neonatal metabolism through breastfeeding. Experimental models that include metabolically normal controls exposed to chronic high levels of a dietary fat have been developed in mice, rats and Japanese macaques [3], [20]–[23]. Obesity prone C57BL/6J mice have proven to be a useful model to distinguish between effects of HF diet and obesity on metabolism [19], [22]. When fed a HF diet, obesity resistant lean (HF-Ln) and obesity prone (HF-Ob) mice can be selected for their differential response in weight gain and adiposity level. Using this model, we have documented that HF-Ln and HF-Ob dams differ from each other, and from low fat (LF) fed dams, in their adaptive responses to lactation, and that these maternal differences were associated with distinct effects on the metabolism and growth rates of their neonates [19].

In the present study, we pursued a molecular understanding of impaired milk fat production in HF-Ob dams. We hypothesized that *de novo* lipogenesis is impaired in HF-Ob mammary glands through the inhibition of key lipogenic enzymes resulting in decreased amounts of *de novo* synthesized fatty acid substrates available for milk lipid production. High dietary fat levels are known to reduce the expression and activity of acetyl-CoA carboxylase-1 (ACC), a prominent regulator of *de novo* fatty acid synthesis [11], [16]. In the mammary gland, ACC expression increases during lactation, presumably in response to the need for increased milk lipid production [16]. In this study, we document differences in the effects of HF diet feeding and obesity on adipose and epithelial compositions, and the levels and activity of ACC and AMPK, in lactating mouse mammary tissue that link impaired milk fat production by obese dams to selective inhibition of epithelial ACC activity.

## Methods

### Feeding Studies and Experimental Design

Twenty female C57BL/6J (C57B6) mice were purchased from Jackson Labs (Bar Harbor, Maine) at 7 weeks of age. After 1 week of acclimation, 5 mice were placed on a defined low fat (LF) diet (68% kcal carbohydrate, 12% kcal fat; #D08032201, Research Diets, New Brunswick NJ) and 15 mice were placed on a defined high fat (HF) diet (34% kcal carbohydrate, 46% kcal fat; #D08032202, Research Diets) for the duration of the study [19]. The LF and HF diets differed only in their relative amounts of carbohydrate (starch) and fat (soy oil) calories. Their total caloric contents and other nutrients and micronutrients were identical. Mice were housed in facilities at the Anschutz Medical Campus’s Center for Comparative Medicine with free access to food and water for the study’s duration (22–24°C; 12∶12 h light-dark cycle). The University of Colorado Anschutz Medical Campus Institutional Animal Care and Use Committee approved all procedures and housing conditions used in the study.

Mice fed the LF diet were classified as LF lean controls and are referred to as the LF-fed group. Following 3 months on the study diets, body composition analyses were performed by quantitative magnetic resonance (QMR; EchoMRI-900 Whole Body Composition Analyzer; Echo Medical Systems, Houston, TX) and mice fed the HF diet were separated into HF lean (HF-Ln) or HF obese (HF-Ob) groups according to their body weights (Figure S1A) and adiposity (body fat) (Figure S1B) as previously described [19]. With this model, we were able to independently address the effects of a HF feeding and obesity on milk composition and mammary gland properties. The effects of HF feeding were determined by averaging results of the two groups of HF fed animals (HF-Ln+HF-Ob) and comparing them to average values of the LF-fed group. The effects of obesity within the context of a HF feeding were determined by comparing average results of the HF-Ln group with those of the HF-Ob group. Following body composition analysis, estrous was induced in mice from each group and they were mated to C57BL/6J males. All mice were housed two per cage and pregnant mice were transferred to individual housing toward the end of pregnancy (P18). After parturition, litters were normalized to 5 pups. Dams and pups were studied at mid-lactation (lactation day 10, (L10)). A comprehensive analysis of maternal and neonatal morphometric and metabolic characteristics have been previously published [19].

### Tissue and Milk Collection

To assess the net retention of *de novo* synthesized lipid in milk and mammary gland tissue an intraperitoneal (I.P.) injection of 100 µCi ^3^H_2_O was given two hours before the beginning of the final dark cycle (L9), allowing time for the tracer to equilibrate with body water [24]. At the end of the subsequent light cycle (L10), lactating dams were anesthetized by I.P. injection of Avertin (250 mg/Kg #T48402 Sigma-Aldrich Corp; St. Louis, MO) and milk letdown was induced with an injection of oxytocin (200 USP units/Kg VetOne, #NDC13985-039-02). Milk was removed under gentle vacuum and stored at −20°C for analysis. Mammary tissue was removed from animals euthanized by cervical dislocation, weighed, and flash-frozen in liquid nitrogen or processed for histology or immunofluorescence analysis.

### Serum Analysis

Colorimetric assays were used to measure serum non-esterified free fatty acids (NEFA) (Wako Chemicals USA, Richmond, VA), glucose and triglycerides (TAGs), (TR15421 and TR22321, respectively, Thermo Fisher Scientific, Waltham, MA). Concentrations of insulin and leptin were simultaneously measured in serum using the Milliplex Map Mouse Serum Adipokine Immunoassay (MADPK-71k-04, Millipore, Billerica MA).

### Milk and Mammary Gland Lipid and Tracer Analysis

To measure total lipid content and radioactively labeled lipids in the milk, lipids were extracted and quantified from 25 µl of milk using the Dole’s extraction mixture (isopropanol: heptane: 1N H_2_SO_4_, 40∶10:1) [25]. Milk lipid extractions were dried down under nitrogen gas and the weight of lipid was recorded. These lipid samples were then suspended in scintillation fluid and the incorporation of ^3^H into lipid was measured with a Beckman LS6500 scintillation counter (Beckman Coulter Inc., Brea CA) using an internal quench correction. Lipids were also extracted from pulverized frozen mammary glands with chloroform-methanol (2∶1, vol/vol) [26]. Phases were separated with the addition of H_2_SO_4_ and centrifugation. The lower phase was collected and allowed to dry to completion under nitrogen gas and suspended in scintillation fluid for ^3^H content analysis. For milk and mammary gland tissue, ^3^H was measured in disintegrations per minute (dpm) and converted to either microcuries (µCi), or nanocuries (nCi), respectively. Mammary gland tracer content is expressed as nCi per gram lipid.

To estimate the total milk lipid and the total *de novo* synthesized lipid that was produced lactating dams over the 24 hr study, the amount of lipid (per ml of milk) measured at the end of the 24 hr study was multiplied by the total volume of milk produced over the 24 hr. Milk volumes were estimated from milk composition and milk energy output as described previously [19].

### Milk Fatty Acid Analysis

Fatty acids in milk were analyzed by gas chromatography at the University of Wisconsin using previously described methods [27]. In some samples, 14∶0 peaks were obscured by, and could not be differentiated from, a large background peak. For these samples, we estimated 14∶0 fatty acid content from the contents of 10∶0 and 12∶0 fatty acids using the approach of Silverman et al [28], which showed that the content of the 14∶0 fatty acids relative to the total pool of 10∶0, 12∶0, and 14∶0 fatty acids in milk lipid was constant (approximately 46%), and independent of dietary fat content. We validated this approach using data from samples free of the artifact peak, showing that the 14∶0 fatty acid content of milk was 43.6±0.1% of the total content of 10∶0,12∶0 and 14∶0 fatty acids.

### Immunoblot Analysis

Frozen mammary samples were homogenized on ice with glass on glass grinding tubes in homogenization buffer (20 mM Tris-HCl, pH 7.4; 150 mM NaCl; 1% NP-40; 20 mM NaF; 2 mM EDTA, pH 8.0; 2.5 mM NaPP; 20 mM b-glycerophosphate; 1% glycerol; ddH2O) plus protease and phosphatase inhibitors (Aprotinin, Leupeptin, Peptstatin, AEBSF, PIC1, PIC2). Protein concentration was measured using the DC Protein Assay Kit II (Bio-Rad # 500-0112). 50 µg amounts of total protein were separated on 7.5% SDS-polyacrylamide gels (Criterion precast gels, BioRAD #345-0005), transferred to a 0.2 µm nitrocellulose membrane (Bio-Rad #162-0112), and probed with the 1/1000 dilutions of rabbit antibodies to: Perilipin 2 (Plin2/ADPH) [29], P-ACCSer79 (Cell Signaling, Danvers, MA), ACC (Cell Signaling), AMPK (Cell Signaling), P-AMPKThr172 (Cell Signaling) and Fatty acid synthase (FAS) (Cell Signaling). Corresponding horseradish-peroxidase-conjugated secondary antibodies (Sigma #A0545) were used at 1/5000 dilution. Bands were detected with SuperSignal West Pico Chemiluminescent substrate (Thermo Scientific #34080) and quantified by chemiluminescence detection using a 16-bit cooled CCD camera and Image Lab ® software (ChemiDoc XRS; Bio-Rad Laboratories, Hercules, CA). Chemiluminescence data were collected at multiple exposure times and were verified to be within the linear response range for all experiments. Immunoblot images used in figures were obtained by exposing immunoblots to X-ray film.

### Histology and Immunohistochemistry

Freshly dissected mammary tissue sections were processed for hematoxylin and eosin (H&E) staining and confocal immunofluorescence microscopy (IF) as described previously [29]. Briefly, H&E Images were captured on an Olympus BX51 microscope equipped with a four megapixel Macrofire digital camera (Optronics, Goleta, CA) using the PictureFrame Application 2.3 (Optronics, Goleta, CA). All images were cropped and assembled using Photoshop CS2 (Adobe Systems Inc. Mountain View, CA).

Sections stained with H&E were also used for quantifying the relative proportions of glandular and adipose compartments in mammary tissue, and for analysis of adipocyte size range. The remainder of the slides were deparaffinized and processed for immunohistochemical analysis [29] **.** The sections were immunolabeled with the specific primary antibodies described above at the following dilutions: Plin2 (1∶100); ACC (1∶100); P-ACC-Ser79 (1∶100); FAS (1∶100). Sections were stained with antibodies to perilipin 1 (#20R-pp004 Fitzgerald, North Acton MA) at a dilution of 1∶100 to identify mammary adipocytes [29] **.** Immunoreactivity was visualized using secondary antibodies conjugated with Alexafluor 488 or Alexafluor 594 (Molecular Probes, Eugene OR) at dilutions of 1∶500 and 1∶250 respectively. Nuclei were stained with 4′,6-diamidino-2-phenylindole (DAPI, Sigma Chemical Company, St Louis, MO).

### Image Analysis

H&E stained sections (5 images from 5 mice/group) were captured using an Aperio Scanscope XT at digital resolutions of 0.43 microns/pixel. Representative sections at 5000×5000 pixel dimensions were exported from the Aperio SVS file format to a Tiff file format for subsequent processing. Exported images were processed inside Adobe Photoshop, highlighting the lumen areas for further analysis. Final analysis on pseudo lumen areas was performed using a custom written plugin for ImageJ (http://rsbweb.nih.gov/ij/docs/faqs.html#cite). Briefly, after excluding the luminal areas, the glandular and adipose compartments in each image were manually identified. The ImageJ plugin described above was then used to calculate the percentages of glandular and adipose tissue in each gland. Averages and SEM values were calculated from 5 randomly chosen mammary gland sections per mouse in treatment groups consisting of 5 mice. Adipocyte size was measured in these sections manually using the measure tool in Photoshop.

Immunofluorescence images were captured on a Nikon Diaphot fluorescence microscope and digitally deconvolved using the No Neighbors algorithm (Slidebook, Denver, CO) as described previously [29]. Specific immunofluorescence intensities were determined in deconvoluted images using the masking and mean intensity functions in Slidebook. Images were converted to TIFF files, and processed by Photoshop (Adobe Systems, Inc., Mountain View, CA) for publication.

### Statistical Analysis

Data were analyzed by analysis of variance (SPSS, version 19.0) with planned comparisons separately examining the effects of HF feeding (LF-fed vs. HF-fed (HF-Ln and HF-Ob)) and obesity in the context of HF feeding (HF-Ln vs. HF-Ob). The data are expressed as means ± SEM for groups of 5 animals comprising at least two separate experiments. Statistical significance was assumed when *p*<0.05.

## Results

### Effects of Obesity on Milk Lipid Production

**Figure 1:**
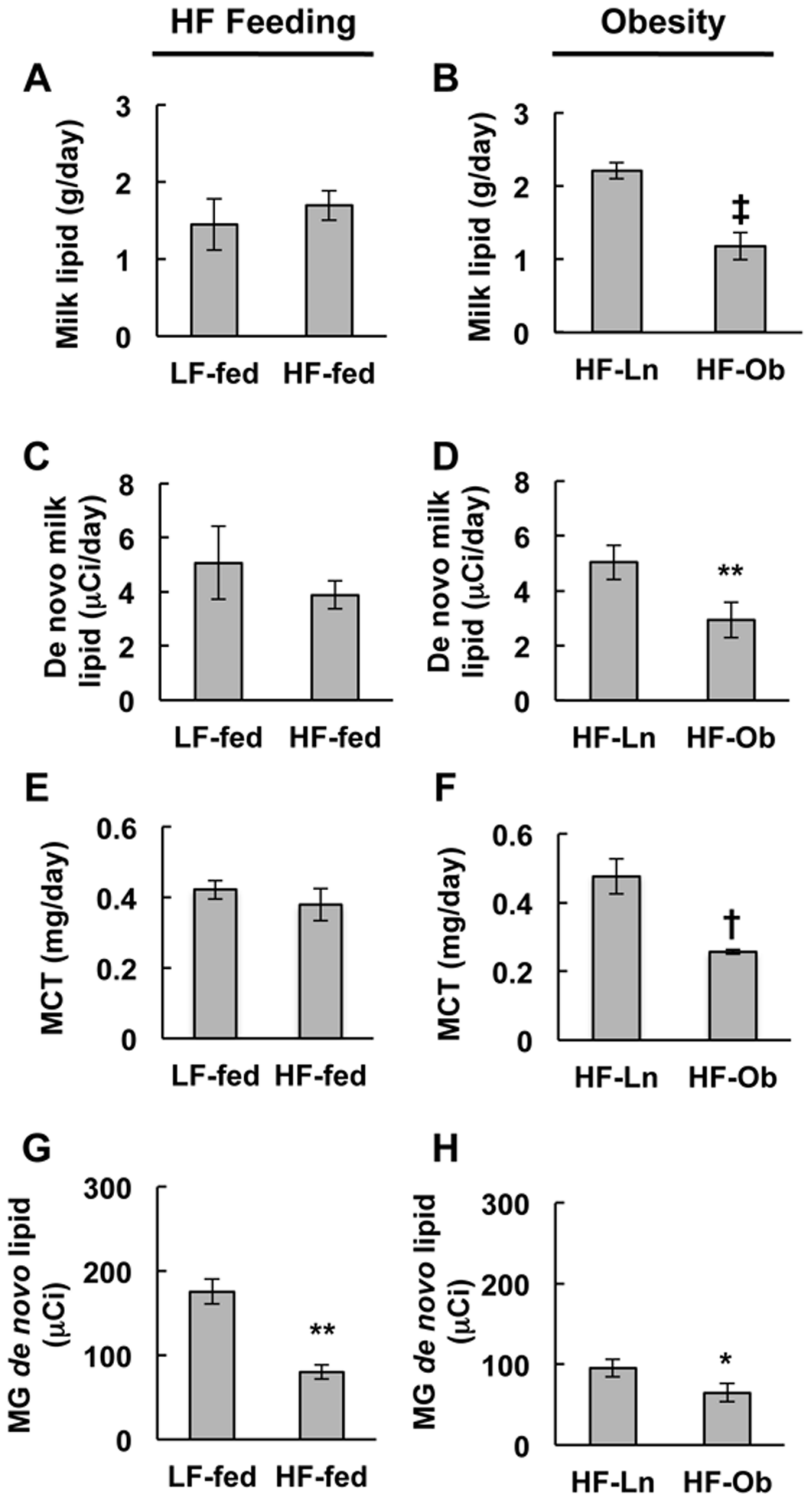
Effects of HF feeding and obesity on milk lipid production. The graphs show the effects of HF feeding (LF-fed vs HF-Ln+HF-Ob) (A, C, E, G) and obesity (B, D, F, H) on 24 h production of total lipids (A, B); de novo synthesized lipids (C, D); and medium chain fatty acids (MCFA) (E, F) in milk of dams at L10; and the content of de novo synthesized lipids in mammary glands of L10 dams (G, H). (A, B) Total milk lipid produced over 24 h. Values were extrapolated from milk lipid measured at the end of the study multiplied by the total milk produced over 24 h as presented previously [19]. (C, D) The incorporation of *de novo* synthesized lipids into milk lipid was estimated from the net retention of ^3^H content within milk lipid over 24 hours, the graph was adapted from Table 2 in reference [19]. (E, F) The quantity of medium chain fatty acids in milk produced over 24 hours, measured via GC mass spectrometry, and expressed as mg/day. (G, H) Mammary gland content of *de novo* synthesized lipids estimated from the retention of ^3^H in the lipid fraction of mammary gland extracts. Values are expressed as nCi/g tissue. All data are expressed as means ± SEM for 5 animals per group. Statistically significant differences are indicate by the following symbols: *p<0.05; **p<0.01; †p<0.003; ‡p<0.002.

We documented previously that inducing obesity prior to pregnancy in female C57B6 mice by feeding them a high fat diet (HF) leads to metabolic alterations during lactation [19]. Additionally, we demonstrated that HF feeding increased the volume of milk consumed by the litters of HF-fed dams, independent of the dam’s adiposity level [19]. Surprisingly, we found that the total amount of milk lipid produced over 24 hrs by HF-Ob dams was only about 50% of that produced by HF-Ln dams (Figure 1B, *p*<0.002), suggesting that maternal obesity impairs milk lipid production. In contrast, 24 hr milk lipid production did not differ between the LF-fed and HF-fed (HF-Ln+HF-Ob) groups (Figure 1A), suggesting that HF feeding per se did not affect milk lipid production. In light of evidence that mid-lactation growth rates of pups nursing from HF-Ob dams were reduced relative to those nursing from HF-Ln dams [19], these observations suggests that maternal obesity may interfere with milk lipid production and impair the ability of HF-Ob dams to meet the energy demands of their litters.

To further evaluate the effects of obesity on milk lipid production, we compared the effects of HF feeding and obesity on the content of *de novo* synthesized lipids in milk and mammary tissue in dams at L10. The total daily production of *de novo* synthesized milk lipids by HF-Ob dams was less than half of that of HF-Ln dams (Figure 1D, *p*<0.01). Consistent with evidence that medium chain fatty acids account for the majority of *de novo* synthesized lipids in milk [30], we found that the total daily production of medium chain fatty acids (MCFA) were also significantly decreased in HF-Ob milk compared to HF-Ln milk (Figure 1F, *p*<0.01). HF feeding was not associated with changes in the amount of *de novo* synthesized milk lipid (Figure 1C) or MCFAs (Figure 1E), suggesting that maternal obesity, not dietary fat content, was responsible for reducing milk fat output. Because reduced milk lipids could result from reduced synthesis and/or reduced secretion, we next investigated the effects HF diet feeding and obesity on mammary *de novo* synthesized lipid levels. Figure 1G and H show that both HF feeding and obesity were associated with lower *de novo* synthesized lipid in mammary tissue (*p*<0.01, *p*<0.05, respectively). Collectively, data in Figure 1 indicate that the reduced lipid levels in milk of obese mice are due to combinations of decreased mammary gland *de novo* lipid synthesis and decreased milk lipid production.

### Milk Fatty Acid Composition

Table 1 depicts the composition of fatty acids in milk from LF-fed, HF-Ln, and HF-Ob dams at mid-lactation as a percentage of total milk lipids. Although we did not observe changes in total MCFA content associated with HF feeding (Figure 1E), there was a 30% reduction in the proportion of MCFAs (C:10-C:14) in milk samples from HF-fed dams (*p*<0.02) compared to LF-fed dams. We also found that the proportions of 16∶0 (23.7±0.9-LF-fed vs.16.4±0.8-HF-fed) and 16∶1 (3.0±0.2-LF-fed vs. 1.2±0.2-HF-fed) fatty acids, which can be obtained exogenously or by *de novo* synthesis [16], in milks of HF-fed dams were significantly reduced (p<0.001). Long chain fatty acids (C:18 or greater) were enriched in milk from HF-fed dams relative to that of LF-fed dams (61.5% vs. 44.3%, respectively; *p*<0.001). Obesity, did not significantly affect the relative fatty acid composition of milk lipids. In combination with total milk lipid data, these findings demonstrate that chronic consumption of a HF diet alters the fatty acid composition of milk lipids but does not affect milk lipid production, whereas maternal obesity reduces total milk lipid levels without markedly affecting their fatty acid composition.

**Table 1:** Milk fatty acid composition.

	LF-fed	HF-fed	*p* value (HF feeding)	HF-Ln	HF-Ob	*p* value (Obesity)
*n*	*5*	*9*		*5*	*4*	
**8∶0**	0.35±0.04	0.3±0.02	0.13	0.3±0.02	0.3±0.06	0.38
**10∶0**	5.8±0.5	4.5±0.24	0.01	4.6±0.4	4.3±0.3	0.59
**11∶0**	0.39±0.09	0.2±0.02	0.01	0.2±0.04	0.2±0.01	0.72
**12∶0**	10.0±0.7	6.8±0.4	0.00	6.8±0.7	6.8±0.2	0.94
**14∶0**	12.3±0.6	8.7±0.5	0.00	8.8±0.9	8.6±0.4	0.83
**14∶1**	0.24±0.03	0.05±0.03	0.00	0.06±0.04	0.05±0.05	0.89
**16∶0**	23.7±0.9	16.4±0.8	0.00	15.7±0.7	17.4±1.9	0.34
**16∶1t**	0.25±0.02	0.2±0.02	0.08	0.18±0.01	0.23±0.03	0.18
**16∶1**	3.0±0.2	1.2±0.2	0.00	1.2±0.1	1.4±0.4	0.48
**17∶1**	0.03±0.04	0.16±0.03	0.02	0.2±0.01	0.1±0.07	0.06
**18∶0**	1.7±0.1	2.1±0.09	0.02	2.1±0.1	2.1±0.2	0.86
**18∶1 oleic**	21.3±1.2	18.0±0.6	0.01	17.8±1.1	18.3±0.4	0.66
**18∶1vaccenic**	3.1±0.2	1.5±0.04	0.00	1.5±0.05	1.6±0.07	0.56
**18∶2**	14.1±0.4	32.4±0.9	0.00	32.9±1.5	31.8±1.4	0.60
**18∶3**	1.2±0.03	2.8±0.09	0.00	2.9±0.1	2.8±0.2	0.52
**18∶3 gamma**	0	0.2±0.05	0.02	0.25±0.08	0.1±0.08	0.23
**20∶1**	0.72±0.09	0.4±0.02	0.00	0.4±0.02	0.4±0.05	0.93
**20∶2**	1.1±0.09	1.6±0.1	0.01	1.6±0.08	1.5±0.2	0.47
**20∶3 gamma**	0.62±0.04	0.9±0.04	0.00	0.9±0.05	0.8±0.06	0.34
**20∶4**	0.5±0.03	0.9±0.04	0.17	0.96±0.08	0.9±0.02	0.38
**22∶1**	0	0.08±0.04	0.17	0.15±0.07	0	0.07
**22∶4**	0	0.15±0.07	0.10	0.22±0.1	0.06±0.07	0.24
**22∶5 n3**	0	0.03±0.03	0.48	0	0.06±0.07	0.29
**22∶6**	0	0.2±0.08	0.10	0.17±0.1	0.2±0.1	0.84
**24∶0/20∶5**	0	0.2±0.07	0.05	0.22±0.1	0.2±0.1	0.81

LF-fed: Low fat lean dams; HF-fed: HF-Ln+HF-Ob dams; HF-Ln: High fat lean dams; HF-Ob: High fat obese dams.

Data expressed as % of the total milk lipid.

The effect of HF feeding = LF-fed vs. HF-fed and the effect of obesity = HF-Ln vs. HF-Ob.

Significance was assumed when p value ≤0.05.

### Mammary Gland Morphology

**Figure 2:**
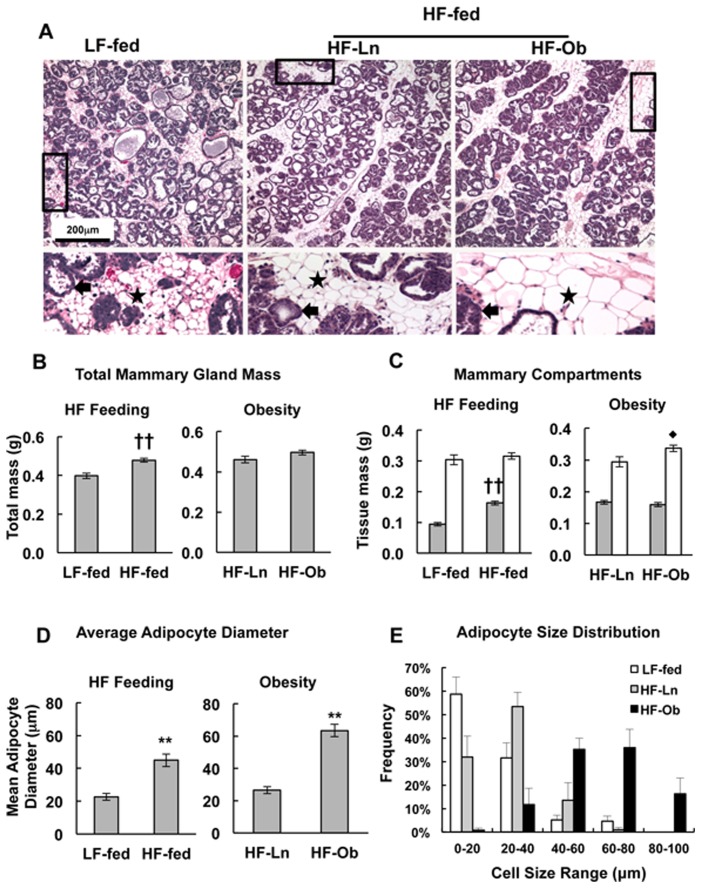
Effects of HF feeding and obesity on mammary gland tissue morphology and composition. Glandular morphologies, and relative epithelial and adipose contents of mammary glands are shown for LF-fed, HF-Ln and HF-Ob dams at L10. (A) Representative images of H&E stained mammary glands. Higher magnification images taken from areas outlined by the black boxes are shown in the lower panels. Arrowheads in higher magnification images indicate mammary epithelium and stars indicate adipocytes. Graphs in B–D compare the effects of HF feeding (LF-fed vs HF-Ln+HF-Ob) and obesity (HF-Ln vs HF-Ob) on mammary gland weights (B), epithelial (white bars) and adipose (grey bars) compositions of mammary glands (C), and adipocyte diameters (D). (E) Shows the size distribution range of mammary gland adipocytes expressed as percentage of adipocytes with diameters in the ranges of 0–20 µm, 20–40 µm, 40–60 µm, 60–80 µm, and 80–100 µm. For panel B, values are means ± SEM for 5 mice per group. For panels C and D, values are means ± SEM, of 5 sections per animal and 5 animals per group. Statistically significant differences are indicate by the following symbols: ♦*p*<0.03; **p<0.01; ††p<0.001.

Diet-induced obesity [11], [12] and abnormalities in mammary gland lipid metabolism have been linked to formation of immature glandular structures in mice [29], [31]–[33]. At the histological level, we did not detect obvious differences in the glandular (alveolar) architectures of lactating LF-fed, HF-Ln or HF-Ob mice (Figure 2A). However, we did find a significant increase in the weights (Figure 2B, *p*≤0.001), the size of the adipose compartment (Figure 2C, *p*<0.001), and the average adipocyte diameter (Figure 2D, *p*<0.01) of mammary glands of HF-fed dams compared to those fed the LF diet. Obesity was not associated with increased mammary gland weight, however HF-Ob dams did have a slight increase in the amount of epithelial tissue compared to HF-Ln dams (Figure 2C, *p*<0.03). Although obesity did not affect the weight-adjusted size of the mammary adipose compartment (Figure 2C), it was associated with a significant increase in the size of adipocytes (p<0.01, Figure 2D). Comparisons of the size distributions of mammary adipocytes from L10 LF-fed, HF-Ln and HF-Ob mice (Figure 2E), shows that both HF feeding and obesity were associated with shifts to cells with larger diameters. Collectively, the results shown in Figure 2 document that HF feeding increases the adipose content and the size of the mammary gland during lactation, whereas obesity appears to independently affect mammary tissue composition by increasing epithelial mass and adipocyte size.

### Effects of Obesity on Glandular Lipid

**Figure 3:**
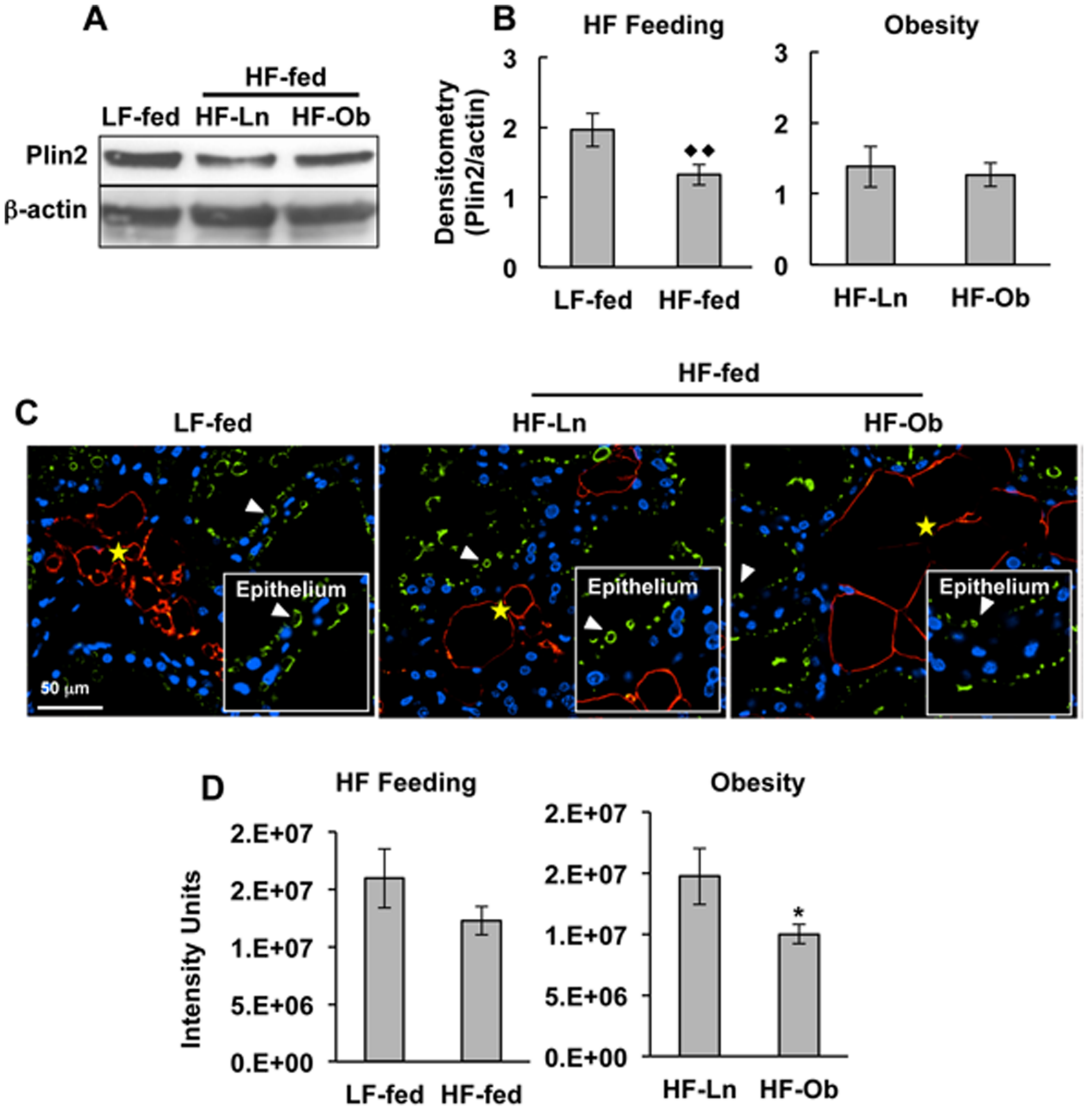
Effects of HF feeding and obesity on perilipin 2 levels in mammary glands of lactating dams. Perilipin 2 levels in mammary glands of LF-fed, HF-Ln and HF-Ob dams at L10. (A) Representative immunoblot images of Plin2 and β-actin in total mammary gland extracts. (B) Chemiluminescence quantification of Plin2 relative to β-actin in mammary gland extracts. The left (HF Feeding) panel compares relative Plin2 levels in mammary gland extracts of LF- and HF-fed dams. The right (Obesity) panel compares relative Plin2 levels in mammary gland extracts of HF-Ln and HF-Ob dams. The values are means ± SEM for Plin2 normalized to β-actin (N = 5). (C) Representative images of mammary gland sections from LF-Fed, HF-Ln and HF-Ob dams at L10 immunostained for Plin2 (green), Plin1 (red, to identify mammary adipose), and DAPI (blue, to identify nuclei). Boxed areas show regions of the mammary epithelium indicated by the white arrows at higher magnification. White arrowheads indicate Plin2 coated CLD in mammary epithelial cells. Yellow stars indicate regions of the mammary gland containing adipose. (D) Quantification of the effects of HF feeding and obesity on Plin2 immunofluorescence in the mammary epithelium. The left (HF Feeding) panel compares average relative Plin2 IF levels in mammary epithelium of LF- and HF-fed (HF-Ln and HF-Ob) dams. The right (Obesity) panel compares average relative Plin2 IF levels in mammary epithelium of HF-Ln and HF-Ob dams. Plin2 IF intensities in mammary alveoli were quantified in 5 randomly chosen sections from each animal and normalized to the percentage of gland within each section. The values are means ± SEM (N = 5). Statistically significant differences are indicate by the following symbols: *p<0.05; ♦♦p<0.02.

The cytoplasmic lipid droplet associate protein, perilipin 2 (Plin2/adipophilin/ADPH), has been implicated in the regulation of milk lipid production and secretion [34]. Previous studies showing that Plin2 is selectively expressed by milk secreting cells in the mouse mammary gland, and that its protein levels correlate with milk lipid production in mice [29], suggest that mammary gland levels of Plin2 may represent a useful surrogate of epithelial cellular lipid content. Interestingly, HF feeding, but not obesity, was associated with significantly lower Plin2 levels in mammary tissue (Figure 3B, *p*<0.02). To address the possibility that the variable amounts of adipose tissue found in mammary glands of lactating LF-fed, HF-Ln and HF-Ob dams confounded Plin2 quantification in whole tissue, we used immunofluorescence (IF) analysis to specifically estimate glandular epithelial Plin2 levels. In agreement with previous studies [29], we found the Plin2 selectively localized to cytoplasmic lipid droplets in milk secreting epithelial cells of the mammary gland (Figure 3C). Quantification of Plin2 IF in mammary epithelial cells is shown in (Figure 3D). Unlike estimates of Plin2 levels in total mammary tissue extracts, we found that Plin2 IF levels in mammary epithelial cells of LF- and HF-fed dams were comparable at L10. In contrast, we found a significant reduction in Plin2 IF levels in mammary epithelial cells of HF-Ob dams compared to HF-Ln dams on L10 (*p*<0.05). These results suggest that obesity significantly reduced Plin2 levels, and by extension the amount of lipid, in the epithelial compartment of lactating mice.

### Effects of Obesity on Mammary Gland Acetyl-CoA Carboxylase-1 (ACC) Protein Levels and Regulation

**Figure 4:**
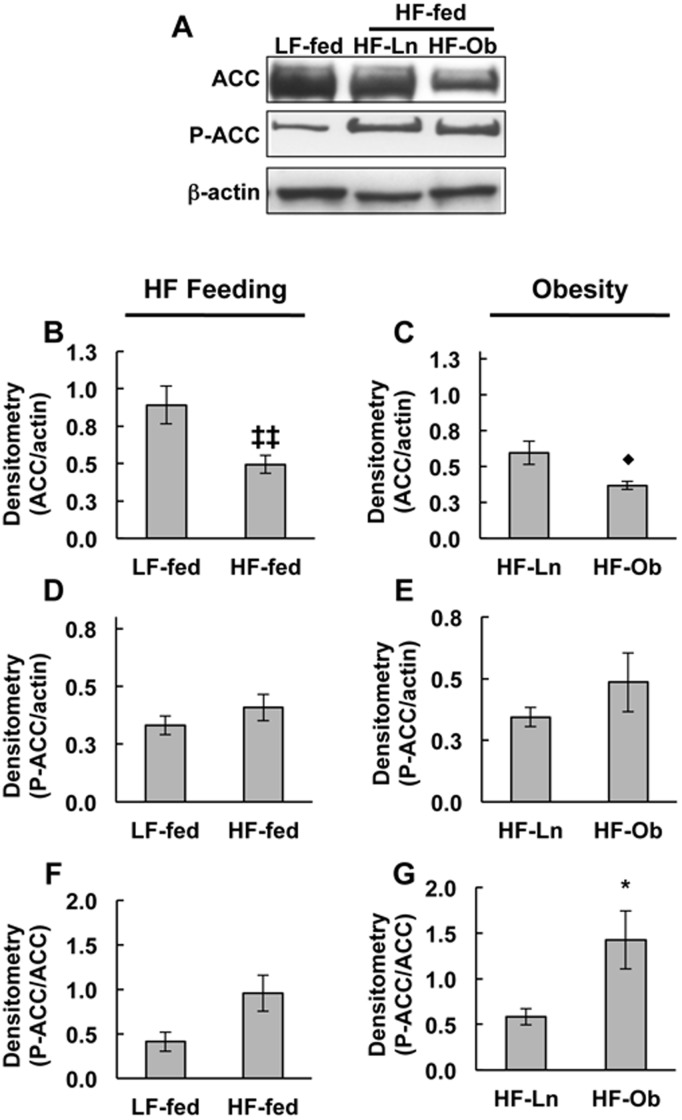
Effects of HF feeding and obesity on ACC and P-ACC levels in mammary glands of lactating dams. ACC and P-ACC levels in mammary glands of LF-fed, HF-Ln and HF-Ob dams at L10. (A) Representative immunoblots of ACC, P-ACC and β-actin in mammary gland extracts. (B–G) Chemiluminescence quantification of ACC (B, C) and P-ACC (D, E) relative to β-actin, and the P-ACC/ACC ratio (F, G) in mammary gland extracts. The values are means ± SEM (N = 5). Panels B, D and F compare the effects of HF feeding (LF-fed vs HF-Ln+HF-Ob) on relative mammary gland levels of ACC (B), P-ACC (D) and P-ACC/ACC (F). Panels C, E and G compare the effects of obesity (HF-Ln vs HF-Ob) on relative mammary gland levels of ACC (C), P-ACC (E) and P-ACC/ACC (G). Statistically significant differences are indicate by the following symbols: *p<0.05; ♦p<0.03; ‡‡p<0.004.

ACC, the rate-limiting enzyme in *de novo* fatty acid synthesis [35], is enriched in lipogenic tissues such as adipose and the lactating mammary gland [36], [37]. Previous studies have reported decreased expression and activity of lipogenic enzymes, including ACC, in lactating mammary glands related HF feeding and/or obesity [11], [15], [38]. However, information about specific effects of HF diet and obesity on ACC levels and activity is limited. As shown in Figures 4, we found that HF feeding (Figure 4B, *p*<0.004) and obesity (Figure 4C, *p*<0.03) were associated with a 60% decrease in the relative levels of ACC in mammary tissue extracts compared to LF-fed and HF-Ln mammary glands, respectively. ACC activity is inhibited by AMP-activated protein kinase (AMPK) dependent phosphorylation of serine at position 79 (P-S79-ACC) [36], [37]. Although the total amount of P-ACC was not significantly effected by HF feeding or obesity (Figure 4D and E), the fraction of inactive enzyme (P-ACC/ACC) was significantly greater in mammary gland extracts of HF-Ob dams relative to extracts of mammary glands from HF-Ln dams (Figure 4G, *p*<0.04). In contrast, we did not observe significant differences in the fraction of inactive ACC between HF-fed and LF-fed mice (Figure 4F). These data document that mammary gland levels of ACC are sensitive to dietary fat, and show that obesity is selectively associated with both a decrease in total ACC levels and a reduction of the fraction of active ACC.

**Figure 5:**
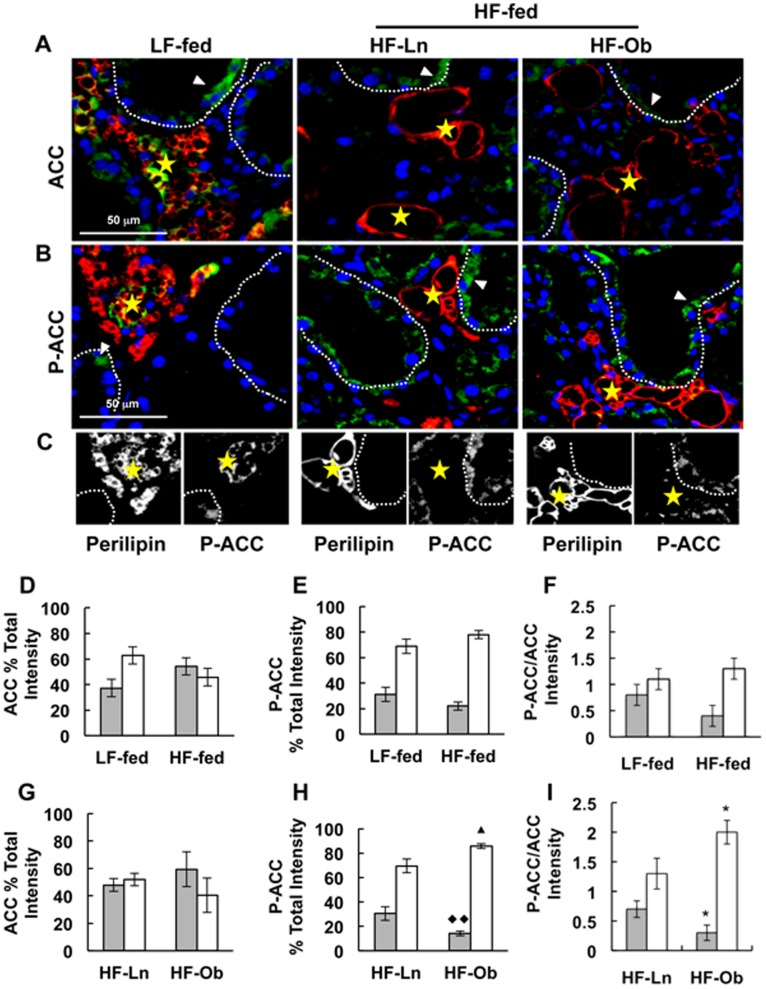
Effects of HF feeding and obesity on P-ACC and ACC in epithelial and adipose compartments. Panels A and B show representative images of mammary gland sections from LF-fed, HF-Ln and HF-Ob dams at L10 immunostained for ACC (green, A) or P-ACC (green, B). Mammary adipose was identified by immunostaining with Plin1 (red) and nuclei were identified by DAPI staining (blue). Panel C shows representative higher (600X) magnification monochrome images of mammary tissue immunostained for P-ACC and Plin1. Dashed white lines outline epithelial compartments; yellow stars indicate areas of adipose, and white arrowheads indicate ACC or P-ACC localization in mammary epithelial cells. Panels D-I show quantification of relative ACC (D, G) and P-ACC (E, H) IF levels and P-ACC/ACC IF ratios (F, I) in epithelial (white bars) and adipose (grey bars) compartments of mammary glands from LF-fed, HF-Ln and HF-Ob dams at L10. Panels D-F compare the effects of HF feeding (LF-fed vs HF-Ln+HF-Ob) on ACC and P-ACC immunofluorescence. Panels G-I compare the effects of obesity (HF-Ln vs HF-Ob) on ACC and P-ACC immunofluorescence. Relative ACC and P-ACC IF levels in each compartment were measured in 5 randomly chosen mammary gland sections and normalized to the respective total ACC or P-ACC IF in each section. The values are means ± SEM for sections from 5 animals per group. Statistically significant differences are indicate by the following symbols: *p<0.05; ♦♦p<0.02; ▴p<0.009.

During lactation, ACC expression and its activity are reported to be reduced in adipose tissue [39], [40] and increased in the mammary epithelium [16], [40], presumably to accommodate the biosynthetic demands of milk lipid production. To determine if these tissue specific responses are altered by HF-feeding or obesity, we used IF microscopy to estimate the relative ACC (Figure 5A) and P-ACC (Figure 5B) levels in the adipose and epithelial compartments. Consistent with our immunoblot results, total mammary gland ACC IF intensity was significantly reduced in HF-fed dams compared to the LF-fed controls (data not shown, *p*<0.004). However, we did not observe significant differences in the proportion of ACC (Figure 5D), P-ACC (Figure 5E), or the fraction of inactive enzyme (P-ACC/ACC, Figure 5F) in either the epithelial or adipose compartments between HF-fed and LF-fed dams.

Obesity was also associated with reduced total mammary gland ACC IF intensity (data not shown, *p*<0.01). We did not find significant effects of obesity on ACC IF levels in the epithelial or the adipose compartments of mammary glands from L10 dams (Figure 5G). However, we did find that obesity was associated with a significant increase in the relative P-ACC IF levels in the epithelial compartment (*p*<0.009), and a significant decrease in P-ACC IF levels in the adipose compartment (*p*<0.02), of mammary glands from dams at L10 (Figure 5H). Comparisons of the P-ACC to ACC IF ratios in HF-Ln and HF-Ob dams in each compartment (Figure 5I) show that the relative levels of inactive (P-ACC) in the epithelial compartments of HF-Ob dams were significantly (∼60%, *p*<0.05) greater than those of HF-Ln dams, whereas the relative levels of P-ACC in the adipose compartment of HF-Ob dams were significantly (∼ 60%, *p*<0.05) less than those of HF-Ln dams. Together, these data suggest that obesity has opposite effects on ACC activities in the epithelium and adipose compartments and are consistent with evidence from our tracer and milk fat analysis studies indicating that obesity impairs *de novo* milk lipid synthesis in lactating dams.

### Obesity does not Affect Mammary Gland Fatty Acid Synthase

To determine if HF feeding or obesity also disrupt fatty acid synthase (FAS), another key player in *de novo* lipogenesis [41], we estimated FAS levels in mammary glands from LF-fed, HF-Ln, and HF-Ob dams (Figure S2). Immunoblot analysis indicated that relative FAS levels in mammary gland extracts of LF-fed, HF-Ln and HF-Ob dams at L10 were similar (Figure S2A). Additionally, although we found that the relative FAS IF intensity in the epithelial compartment was greater than that in the adipose compartment (Figure S2B), we did not detect significant effects of HF feeding or obesity on FAS IF levels in either compartment (Figure S2C). Thus, unlike ACC levels, mammary gland FAS levels in our model do not appear to be sensitive HF feeding or obesity. Furthermore, decreases in FAS do not appear to contribute to the effects of obesity on milk lipid levels, or mammary gland *de novo* lipid synthesis.

### Obesity Increases Mammary Gland AMPK Activity

**Figure 6:**
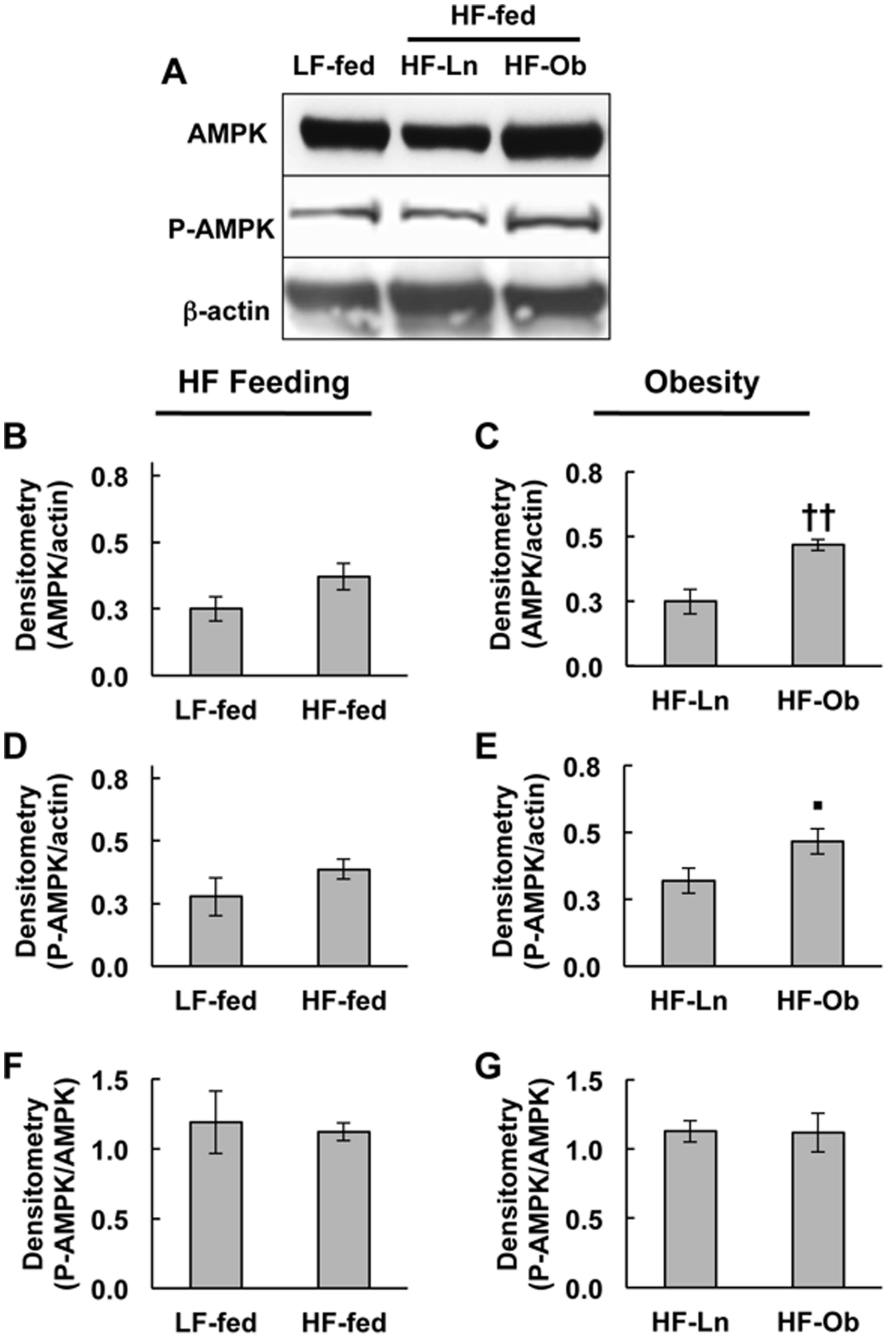
Effects of HF feeding and obesity on AMPK and P-AMPK levels in mammary glands of lactating dams. (A) Representative immunoblots of AMPK, T172-P-AMPK (P-AMPK) and β-actin in extracts of mammary glands from LF-fed, HF-Ln and HF-Ob dams at L10. (B–G) Chemiluminescence quantification of AMPK (B, C) and P-AMPK (D, E) relative to β-actin, and the P-AMPK/AMPK ratio (F, G) in mammary gland extracts. Panels B, D, and F compare the effects of HF feeding (LF-fed vs HF-Ln+HF-Ob) on relative mammary gland levels of AMPK (B), P-AMPK (D) and P-AMPK/AMPK (F). Panels C, E, and G compare the effects of obesity (HF-Ln vs HF-Ob) on relative mammary gland levels of AMPK (C), P-AMPK (E) and P-AMPK/AMPK (G). All values are means ± SEM. N = 5 animals per group. Statistically significant differences are indicate by the following symbols: ▪*p*<0.04; ††p<0.001.

AMPK, a primary kinase regulator of ACC in liver and skeletal muscle, is responsible for decreasing ACC activity and inhibiting fatty acid synthesis in these tissues [42], [43]. To determine if ACC phosphorylation in mammary glands of obese dams was associated with AMPK activation, we compared the relative levels of total AMPK and threonine 172-phosphorylated (activated) AMPK (P-AMPK) in extracts of mammary glands from LF-fed, HF-Ln and HF-Ob dams (Figure 6). Compared to LF-fed dams, we did not detect differences in the relative levels of AMPK and P-AMPK in mammary gland extracts of HF fed dams (Figures 6B, D, and F). In contrast, the relative levels of AMPK (Figure 6C, *p*<0.001) and P-AMPK (Figure 6E, *p*<0.04) in mammary gland extracts of HF-Ob dams were significantly elevated over their levels in mammary gland extracts of HF-Ln dams. We did not detect significant differences in the P-AMPK/AMPK ratios in mammary gland extracts of LF- compared to HF-fed dams (Figure 6F) or in extracts of HF-Ln compared to HF-Ob dams (Figure 6G), suggesting that the relative activation state of AMPK in mammary tissue is not influenced by dietary fat content or obesity. Collectively, these data are consistent with the possibility that elevated AMPK activity, due to increased AMPK protein levels, may contribute to the increased phosphorylation and inactivation of ACC observed in the mammary epithelium of HF-Ob dams.

## Discussion

The energetic demands of lactation induce coordinated changes in the metabolic properties of the mammary gland and other metabolic tissues to provide the necessary energy and substrates for milk production [44]. Previous data suggest that in mice, obesity alters maternal metabolism resulting in production of lipid poor milk that is associated with the inability of obese dams to meet the energy requirements for offspring growth during the neonatal period [19]. Our study now provides evidence that production of lipid poor milk by obese dams results, in part, from selective impairment of ACC activity, and the concomitant decrease in fatty acid synthesis, in mammary epithelial cells. Moreover, because mammary epithelial cells uniquely synthesize only MCFA [45], our data further suggest that the combined effects of reduced milk lipid secretion and impaired fatty acid synthesis are likely to be primary contributors to reduced MCFA production in milk of obese dams.

In humans impaired MCFA oxidation is associated with neonatal metabolic disorders and elevated morbidity [46], and animal model studies have demonstrated that MCFA are more readily absorbed by the gut and transported to the liver for oxidation than long chain fatty acids (LCFA) [47], [48]. These observations indicate that MCFA may be the preferred energy source for growth in the developing neonate, and suggest that reduced MCFA consumption would alter neonatal metabolic properties and lead to impaired growth. Although additional studies are needed to establish the functional importance of MCFA in neonatal growth and metabolism, our data are consistent with the possibility that decreased neonatal MCFA consumption, due to impaired mammary gland synthesis and milk production, contributes to impaired β-oxidation and neonatal growth rates observed in off-spring of HF-Ob dams [19]. It remains to be determined to what extent maternal obesity affects MCFA in human milk and if altered milk MCFA affect neonatal metabolism and growth of human infants. However, evidence that MCFA consumption is associated with improved weight control and satiety regulation [49], and that the composition of fats ingested early in life is a potential determinant of subsequent childhood overweight and obesity risk [50], suggest that MCFA in breast milk may a modifiable factor that influences human neonatal metabolism and health outcomes.

HF diets are known to be associated with reductions in the concentration of *de novo* synthesized lipids in milk [14]. This is a logical adaptive response to the increased availability of preformed fats and limited availability of precursors for *de novo* lipid synthesis. A similar argument explains the increase in long-chain fatty acids in the milk of HF fed dams relative to those fed the LF diet. Beyond substrate availability, the regulatory mechanisms responsible for this shift in fuel utilization are presumably multifactorial, and likely include transcriptional and posttranslational regulation of enzymes involved in *de novo* fatty acid synthesis [16]. Our data from HF-Ln dams are consistent with this concept. Importantly, our study provides the first evidence suggesting that maternal obesity may impair *de novo* lipid synthesis in the lactating mammary gland by AMPK mediated phosphorylation and inhibition of ACC. Collectively, our data suggest that the marked reduction in milk lipid production observed in obese dams is likely due to the inhibition of *de novo* milk lipid synthesis, which is mediated by the compound actions of high dietary fat-dependent reduction of mammary gland ACC levels, and obesity-dependent inhibition of mammary gland ACC activity through the activation of AMPK.

Interestingly, we did not observe decreased amounts of FAS in mammary glands of dams fed a HF-diet, as was recently reported by Rudolph et al [16]. The Rudolph et al study utilized a different mouse strain (FVB), and their dams were fed a HF-diet for only 5 days, beginning on day 17 of pregnancy. At present, it is unclear whether such differences contributed to the discrepancy in the effects of HF-feeding on mammary gland FAS levels. Nevertheless, the absence of significant effects of HF-feeding or obesity on mammary gland FAS levels, indicate that differences in FAS levels are not part of the mechanism by which obesity affects *de novo* milk lipid production in our study. Because ACC catalyzes formation of malonyl-CoA, which along with acetyl-CoA is one of the two substrates used by FAS for fatty acid synthesis [51], our data suggest that mechanism by which obesity impairs *de novo* lipogenesis in the lactating mammary gland is by reducing malonyl-CoA production through posttranslational inhibition of ACC.

As a master regulator of cellular energy and homeostasis, AMPK activation is known to increase catabolic activity and decrease anabolic processes, such as *de novo* lipogenesis and triglyceride synthesis in many metabolic tissues [52], [53]. During lactation, the mammary gland is one of the most anabolically active organs in the body [38], [54], and consequently it may be particularly sensitive to the catabolic regulation of AMPK. Consistent with this assertion, McFadden et. al. showed that direct AMPK activation suppressed *de novo* lipid synthesis and triglyceride formation in mammary epithelial cells [55]. Although our data support the conclusion that elevated AMPK activity is associated with impaired *de novo* lipogenesis in mammary epithelium of obese mice, additional functional studies, using pharmacological and/or genetic approaches to disrupt mammary gland AMPK activity, are required to demonstrate a direct relationship between these two observations and validate the physiological importance of AMPK in regulating *de novo* lipid synthesis in the lactating mammary gland.

The factors activating AMPK in mammary glands of obese dams are unclear at present. Diet-induced obesity has been shown to impair AMPK regulation by leptin, insulin, and sympathetic neural efferent’s in other tissues [56], and aspects of AMPK function appear to be restored with weight loss [57], [58]. However, leptin and free fatty acids, which are elevated in obese individuals [59], [60] and tend to be higher in lactating obese mice (*unpublished data*), have also been shown to activate AMPK by stimulating its phosphorylation [61], [62]. Finally, the elevated AMPK we observe in glands of HF-Ob dams may reflect an energy-deprived state of cells that are incapable of producing the energy to meet the costs associated with milk production. Consistent with this concept, we have previously documented that HF-Ob dams traffic a greater proportion of consumed lipids to their liver and visceral adipose, potentially leaving the mammary gland deprived of energy [19]. However, clarification of the contribution of these and other mechanisms behind obesity’s effects on mammary AMPK during lactation will require additional studies.

Finally, mammary adipose tissue undergoes marked depletion of its lipid stores in response to lactation [14], [29], [63] at a time when there is a corresponding increase in milk lipid synthesis, suggesting that functional linkages exist between adipose and epithelial compartments during lactation. Our observations that the adipose content of mammary glands is elevated by HF feeding and obesity, suggest that high dietary fat consumption may interfere with these linkages, impairing lactation-dependent depletion of mammary adipose lipid stores. Interestingly, we found that obesity and HF diet produced distinct effects on the size of mammary adipocytes, suggesting differences in their actions. The attenuation of adipose depletion associated with HF feeding in lean dams occurs in the context of smaller adipocytes. Thus, our observations in HF-Ln dams are consistent with the concept that elevated dietary fat reduces the requirement for mobilization of stored fat to provide substrates for milk lipid synthesis [14], [15], [64]. Conversely, large adipocytes associated with obesity are less metabolically active and resistant to lipolytic stimulation [65], [66]. Consistent with this concept, our data suggests that the large adipocytes observed in HF-Ob dams might be resistant to signals that promote the adipose lipid mobilization during lactation. Because it is thought that such mobilization contributes substrates for milk lipid production, our data provide evidence that impaired adipose lipid mobilization may be and additional mechanism by which obesity contributes to reduced levels of milk lipid.

The limited degree of ACC phosphorylation in the adipose compartment of obese mice stands in stark contrast to that observed in the epithelial compartment. Differences in the compartmental regulation of ACC may promote trafficking of excess nutrients toward adipocytes, and away from milk producing epithelial cells. In agreement with this concept, we showed previously that lipid trafficking to visceral adipose tissues was increased by obesity [19]. Taken together, these observations implicate a failure to mobilize endogenous energy and traffic ingested nutrients to milk production as an additional contributor to reduced milk lipid levels in obese dams.

In conclusion, our data identify both impaired *de novo* fatty acid synthesis related to AMPK inhibition of ACC activity, and disrupted adipose lipid mobilization as possible mechanisms underlying defects in milk lipid content of obese dams. Further work will be required to understand the contributions of adipose lipid mobilization to formation of milk lipids, and to delineate the mechanism(s) by which AMPK is activated by obesity. Importantly, the concept that AMPK activity is directly responsible for obesity related decreases in ACC activity and fatty acid synthesis in mammary epithelial cells needs to be formally tested before it can be concluded that this pathway is a primary regulator of the effects of obesity on mammary gland function. Nevertheless, our findings that AMPK-activation is associated with decreased *de novo* lipid synthesis, and reduced lipid levels in mammary epithelial cells, agree with the known ability of AMPK activation to reduce cellular lipid levels by inhibiting *de novo* lipogenesis and stimulating lipid oxidation in other metabolic tissues, such as liver or skeletal muscle [62]. Finally, although AMPK activation is generally believed to be advantageous in the treatment of metabolic disorders, our data suggest that increased AMPK activation in mammary glands of obese mice is associated with impaired milk lipid synthesis and formation of calorically depleted milk. Thus our study raises questions about possible disadvantageous effects of AMPK-targeted agents used for treating metabolic disorders on milk composition and subsequent infant nutrition for breastfeeding women.

## Supporting Information

Figure S1 **Effects of HF-feeding on pre-pregnancy body weight gains and the body compositions of dams at L10.** Eight-week old mice were placed on a low fat (LF) diet (12% kcal fat; n = 5) or a high fat (HF) diet (46% kcal fat; n = 15) for the duration of the study. (A) Individual body weights were determined weekly for 11 weeks prior to mating. Based on body weight gain over this time, the HF-fed group was separated into mice that gained weight in response to the HF diet (HF-Ob; n = 5) and mice that were resistant to the HF diet (HF-Ln; n = 5). Five mice that showed an “in between” phenotype were removed from the study. (B) Body composition was determined on L10 using quantitative magnetic resonance. Values are expressed as mean ± SEM. Statistically significant differences are indicate by *p<0.05.
(TIF)

Figure S2 **Effects of HF-feeding and obesity on mammary gland levels and localization of FAS.** (A) Representative immunoblots of FAS and β-actin in extracts of mammary glands from LF-fed, HF-Ln and HF-Ob dams at L10. (B) Chemiluminescence quantification of FAS relative to β-actin in mammary gland extracts. The left (HF Feeding) panel compares relative FAS levels mammary gland extracts of LF- and HF-fed (HF-Ln+HF-Ob) dams. The right (Obesity) panel compares relative FAS levels in mammary gland extracts of HF-Ln and HF-Ob dams. The values are means ± SEM for FAS normalized to β-actin (N = 5). (C) Shows representative images of mammary glands from LF-fed, HF-Ln and HF-Ob dams at L10 immunostained for FAS (green), Plin1 (red, to identify adipose) and DAPI (blue, to identify nuclei). White arrowheads indicate FAS in mammary epithelial cells; dashed white lines outline epithelial compartments, yellow stars indicate areas of adipose. (D) Quantification of the effects of HF feeding and obesity on relative FAS IF levels in mammary epithelial (white bars) and adipose (grey bars) compartments of dams at L10. The left panel (HF Feeding) compares average relative FAS IF levels in the epithelial and adipose compartments of LF-fed dams with average FAS IF levels of HF fed (HF-Ln and HF-Ob) dams. The right panel (Obesity) compares average relative FAS IF levels in the epithelial and adipose compartments of HF-Ln and HF-Ob dams. FAS IF levels were measured in the respective glandular or adipose compartments in 5 randomly chosen mammary gland sections from each animal and normalized to total FAS IF levels in the respective section. The values are means ± SEM for 5 animals per group.
(TIF)
