# Supplementary figures and images for: Maternal Obesity Reduces Milk Lipid Production in Lactating Mice by Inhibiting Acetyl-CoA Carboxylase and Impairing Fatty Acid Synthesis
Source: PLoS One. 2014 May 21;9(5):e98066. doi: 10.1371/journal.pone.0098066 (PMC4029960; doi:10.1371/journal.pone.0098066)

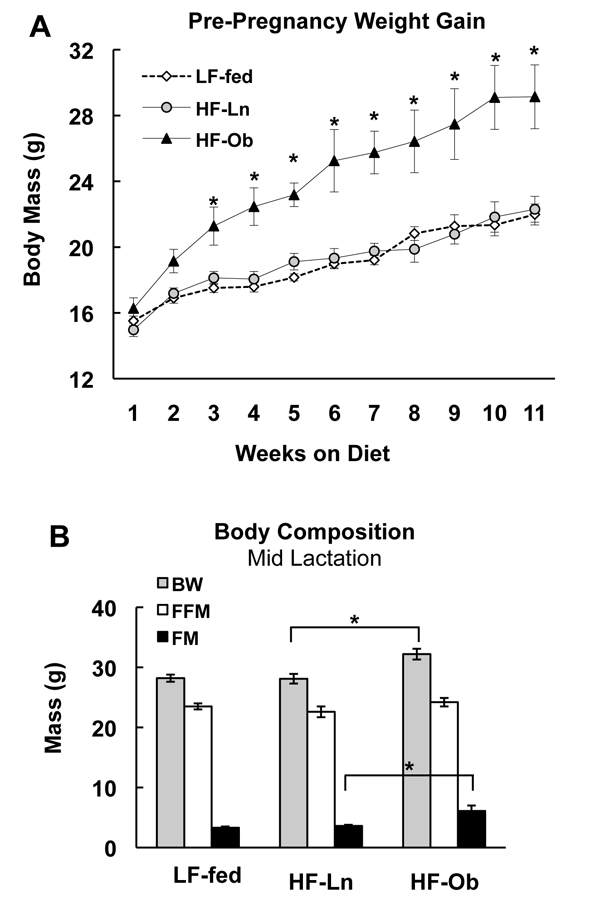

Supplement: Figure S1 — Effects of HF-feeding on pre-pregnancy body weight gains and the body compositions of dams at L10. Eight-week old mice were placed on a low fat (LF) diet (12% kcal fat; n = 5) or a high fat (HF) diet (46% kcal fat; n = 15) for the duration of the study. (A) Individual body weights were determined weekly for 11 weeks prior to mating. Based on body weight gain over this time, the HF-fed group was separated into mice that gained weight in response to the HF diet (HF-Ob; n = 5) and mice that were resistant to the HF diet (HF-Ln; n = 5). Five mice that showed an “in between” phenotype were removed from the study. (B) Body composition was determined on L10 using quantitative magnetic resonance. Values are expressed as mean ± SEM. Statistically significant differences are indicate by *p<0.05. (TIF) [file pone.0098066.s001.tif]

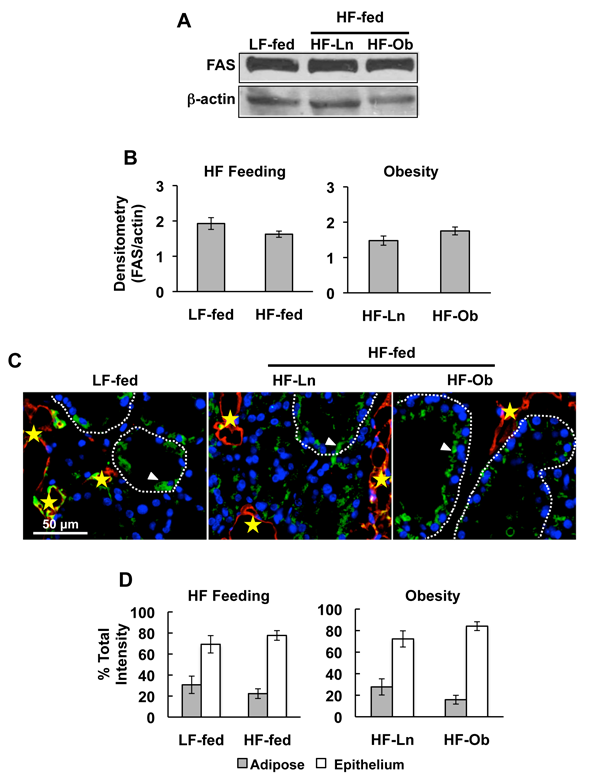

Supplement: Figure S2 — Effects of HF-feeding and obesity on mammary gland levels and localization of FAS. (A) Representative immunoblots of FAS and β-actin in extracts of mammary glands from LF-fed, HF-Ln and HF-Ob dams at L10. (B) Chemiluminescence quantification of FAS relative to β-actin in mammary gland extracts. The left (HF Feeding) panel compares relative FAS levels mammary gland extracts of LF- and HF-fed (HF-Ln+HF-Ob) dams. The right (Obesity) panel compares relative FAS levels in mammary gland extracts of HF-Ln and HF-Ob dams. The values are means ± SEM for FAS normalized to β-actin (N = 5). (C) Shows representative images of mammary glands from LF-fed, HF-Ln and HF-Ob dams at L10 immunostained for FAS (green), Plin1 (red, to identify adipose) and DAPI (blue, to identify nuclei). White arrowheads indicate FAS in mammary epithelial cells; dashed white lines outline epithelial compartments, yellow stars indicate areas of adipose. (D) Quantification of the effects of HF feeding and obesity on relative FAS IF levels in mammary epithelial (white bars) and adipose (grey bars) compartments of dams at L10. The left panel (HF Feeding) compares average relative FAS IF levels in the epithelial and adipose compartments of LF-fed dams with average FAS IF levels of HF fed (HF-Ln and HF-Ob) dams. The right panel (Obesity) compares average relative FAS IF levels in the epithelial and adipose compartments of HF-Ln and HF-Ob dams. FAS IF levels were measured in the respective glandular or adipose compartments in 5 randomly chosen mammary gland sections from each animal and normalized to total FAS IF levels in the respective section. The values are means ± SEM for 5 animals per group. (TIF) [file pone.0098066.s002.tif]
